# Clinical study of carotid superb microvascular imaging in evaluating the activity of Takayasu’s arteritis

**DOI:** 10.3389/fcvm.2023.1051862

**Published:** 2023-03-06

**Authors:** Feng-Ju Liu, Wei-Ping Ci, Yi Cheng

**Affiliations:** ^1^Department of Ultrasound, Beijing Anzhen Hospital, Beijing Institute of Heart, Lung, and Blood Vessel Diseases, Capital Medical University, Beijing, China; ^2^Department of Rheumatology and Immunology, Beijing Anzhen Hospital, Beijing Institute of Heart, Lung, and Blood Vessel Diseases, Capital Medical University, Beijing, China

**Keywords:** Takayasu's arteritis, superb microvascular imaging, Doppler ultrasound, clinical study, carotid superb microvascular imaging

## Abstract

**Objective:**

The goal of this study is to use superb microvascular imaging (SMI) to observe neovascularization in the carotid vessel wall to identify potential Takayasu’s arteritis (TAK) inflammation markers.

**Methods:**

Bilateral carotid arteries from 96 patients with TAK were imaged by a Doppler ultrasound and SMI. The one-way analysis of variance (ANOVA) was used to document significant differences between the activity and inactivity stages of TAK and the factors closely related to its activity in the binary logistics regression equation. Clinical and laboratory data included age, gender, duration of disease, treatment history, NIH score, erythrocyte sedimentation rate (ESR), and high-sensitivity C-reactive protein. Imaging data included the arterial wall thickness, degree of lesion, SMI grade, and arterial aneurysm formation.

**Results:**

There were 45 patients in the active TAK stage and 51 in the inactive stage. The one-way ANOVA showed significant differences in SMI (*p* = 0.001) and ESR (*p* = 0.022) between the active and inactive groups. The binary logistics regression analysis showed that SMI was an independent risk factor for TAK activity (B = −1.505, S.E = 0.340, Wald = 19.528, OR = 0.222 95%, CI = 0.114–0.433, *p* < 0.01). Using SMI G1 or G2 as the cutoff values for the diagnosis of active TAK, the positive predictive value, sensitivity, and specificity were 60 and 86%, 84% and 56%, and 54% and 92%, respectively.

**Conclusion:**

The SMI grade is a potential marker of disease activity in patients with TAK.

## Introduction

Takayasu’s arteritis (TAK) is a chronic, nonspecific, large vessel vasculitis primarily affecting the aorta and its major branches, and it occurs relatively often in younger women (age < 50 years) (1, 2). Contrast-enhanced ultrasound (CEUS) can semi-quantitatively evaluate the formation of carotid wall angiogenesis. Therefore, it has been proposed that ultrasonographic imaging of microbubble development in thickened carotid walls may be useful for the assessment of carotid inflammation in TAK (3, 4). Recently, Toshiba developed an innovative Doppler ultrasound technology called superb microvascular imaging (SMI) using the Aplio™ 500 ultrasound system (Toshiba Medical Systems Corporation, Tochigi, Japan), enabling the visualization of slow-flow vessels without the need for a contrast medium (5, 6). Previous work has shown that SMI is well correlated and consistent with CEUS in its ability to detect plaque neovascularization (7–9). Microvascular hyperplasia is the most characteristic pathological change in the active TAK. Therefore, the goal of this study is to use contrast-free SMI imaging to observe tiny blood vessels that proliferate in the vessel wall to identify potential imaging markers reflecting TAK active inflammation.

## Materials and methods

### Study participants

We prospectively studied patients with TAK who were in-patients of the rheumatology department of the Beijing Anzhen Hospital from October 2015 to September 2021. The study was approved by the institutional review board and all enrolled patients provided written informed consent. A total of 96 patients with a clinical diagnosis of TAK with carotid artery involvement, based on physical examinations, laboratory studies, and surgical pathologic examinations, were evaluated using vascular sonography. Some patients underwent computed tomographic angiography, magnetic resonance imaging (MRI), and other imaging examinations. The diagnosis of TAK was made using the 1990 standard for TAK from the American College of Rheumatology (10), the Antineutrophil Cytoplasmic Antibody 2012 Workshop on Takayasu Arteritis (1), and the revised nomenclature for vasculitis from the 2012 International Chapel Hill Consensus Conference (11).

### Carotid ultrasonography

All patients received standard vascular ultrasound examination of the bilateral subclavian, carotid, and vertebral arteries within 24 h of admission using a 4–9 MHz linear-array transducer with SMI capabilities (Aplio 500, Toshiba Medical, Tokyo, Japan). Measurements were recorded by two well-trained technicians who were blinded to all information about the study participants.

### The SMI classification

The SMI classification was made based on the CEUS classification standard previously described by Deyama et al. (12). Neovascularization within the carotid wall was identified as continuous or discontinuous short lines, dots, or short rod-shaped, medium to strong, echogenic and was graded as G0 = no visible short lines, dots, or short rod-shaped, medium to strong, echogenic within the wall; G1 = moderate visible short lines, dots, or short rod-shaped, medium to strong, echogenic within the wall; G2 = extensive visible short lines, dots or short rod-shaped, medium to strong, echogenic within the wall. For each patient, the carotid wall with the highest grade of neovascularization was selected for the majority of the analyses.

### Clinical data collection

#### Baseline date

Clinical data included age, gender, duration of disease, pharmacologic treatment history (hormones and/or immunosuppressants), and the NIH score. Ultrasound data included the SMI grade, thickness of the involved arterial wall (The typical “macaroni” sign in transverse section consists of three layers of “strong - medium (low) - strong” echo. The vertical distance between the outer edges of two strong echo lines at the thickest part of the vessel wall was measured), and degree of lesion (1 = concentric thickening of the carotid wall, 2 = stenosis of lumen, 3 = occlusion of the lumen).

#### Laboratory data

The erythrocyte sedimentation rate (ESR) and high-sensitivity C-reactive protein (hsCRP) levels were measured and recorded during the ultrasound examination. ESR was measured using the modified Westergren method and the hsCRP levels were measured using immunofluorescence turbidimetry (DiaSys Diagnostic Systems GmbH, Alte Strasse 9, 65,558, Holzheim, Germany). The normal range for ESR was defined as 0–15 mm/h for male patients and 0–20 mm/h for female patients. Elevated hsCRP was defined as >5 mg/L.

#### Criteria of active TAK

The criteria for patients with TAK in the active stage were as follows (13): among patients with TAK, two or more of the following four factors indicated that the disease had aggravated and was active: (1) systemic manifestations, such as fever and joint and muscle involvement (except for other reasons); (2) manifestations of vascular inflammation or ischemia, such as intermittent claudication, diminished or absent pulse, vascular murmur, vascular pain, or bilateral upper or lower limb blood pressure asymmetry; (3) increased ESR and/or hsCRP levels, excluding active infection; (4) typical angiographic abnormality.

#### NIH score

The NIH scores were based on the National Institutes of Health (NIH) criteria (13). The disease status of patients with TAK was determined to be a score of 1 on the NIH scale if they had one of the features of the criteria, and it was determined to be a score of 2 on the NIH scale if they met two of the above criteria, and so on in the same way.

### Statistical methods

The statistical software package SPSS 22.0 (IBM, Armonk, United States) was used for data analysis. The data were summarized as mean ± standard deviation (SD) and median (interquartile range) for continuous variables and the number of subjects (percent) for categorical variables. The one-way analysis of variance (ANOVA) was used to record the significant differences between the two groups and the factors closely related to TAK activity into the logistics regression equation. The stepwise forward method was used to screen the variables, and *p* < 0.05 was considered statistically significant.

## Results

### Patient characteristics

There were 45 patients in the active TAK stage and 51 in the inactive stage. Those 96 patients with TAK underwent ultrasonic scanning, and the findings were compared to clinical characteristics and laboratory values (Table 1). Mean of ESR were higher in the active TAK compared to the inactive TAK group [13.11 (SD: 16.72) vs. 6.84 (SD: 4.44), *p* = 0.022, respectively]. Median SMI grades were higher in the active TAK compared to the inactive TAK group [2 (IQR: 1–2) vs. 0 (IQR: 0–1), *p* = 0.001, respectively]. The binary logistics regression analysis showed that SMI was an independent risk factor for TAK activity (B = 1.564, S.E = 0.355, Wald = 19.436, OR = 4.778, 95% CI = 2.384–9.576, *p* < 0.01). The SMI G1 or G2 were used as the cut-off values for the diagnosis of the active TAK stage, and the diagnostic efficacy is shown in Table 2.

**Table 1:** Clinical data of Takayasu’s arteritis patients *n* = 96.

Variable	Value
Gender (female,*n*%)	91,95%
Age (year), Range	(36 ± 12)^**^, 15–59
SMI Grade (*n*,%)	0(35,37%) 1(32,33%) 2(29,30%)
TH (*n*,%)	+ (32,33%) − (64,67%)
DL Grade (*n*,%)	1(17,18%) 2(33,34%) 3(46,48%)
hsCRP (mg/L)	0.85(1.52 4.02)^*^
ESR (mm/h)	6 (2 11)^*^
DD (month)	12 (3.5168)^*^
NIH (grade), Range	1.48 ± 0.91^**^, 0–4
WT (cm), Range	0.23 ± 0.08^**^, 0.10–0.62

^*^Medians (interquartile range); ^**^mean ± standard deviation; SMI, Superb Micro-Vascular Imaging; TH, history of pharmacologic treatment; “+,” have been treated; “−,” not treated; DL, degree of lesion (1 = wall thickening; 2 = lumen stenosis; 3 = lumen occlusion); hsCPR, high-sensitivity C-reactive protein; ESR, erythrocyte sedimentation rate; DD, Duration of disease; NIH, National Institute of Health score; WT, Vascular wall thickening.

**Table 2:** Efficacy of SMI grade in the diagnosis of Takayasu’s arteritis.

	PPV	NPV	Sensitivity	Specificity
SMI G1	60%	80%	84%	54%
SMI G2	86%	70%	56%	92%

PPV, Positive predictive value; NPV, negative predictive value.

### Imaging characteristics of carotid ultrasound

**Figure 1:**
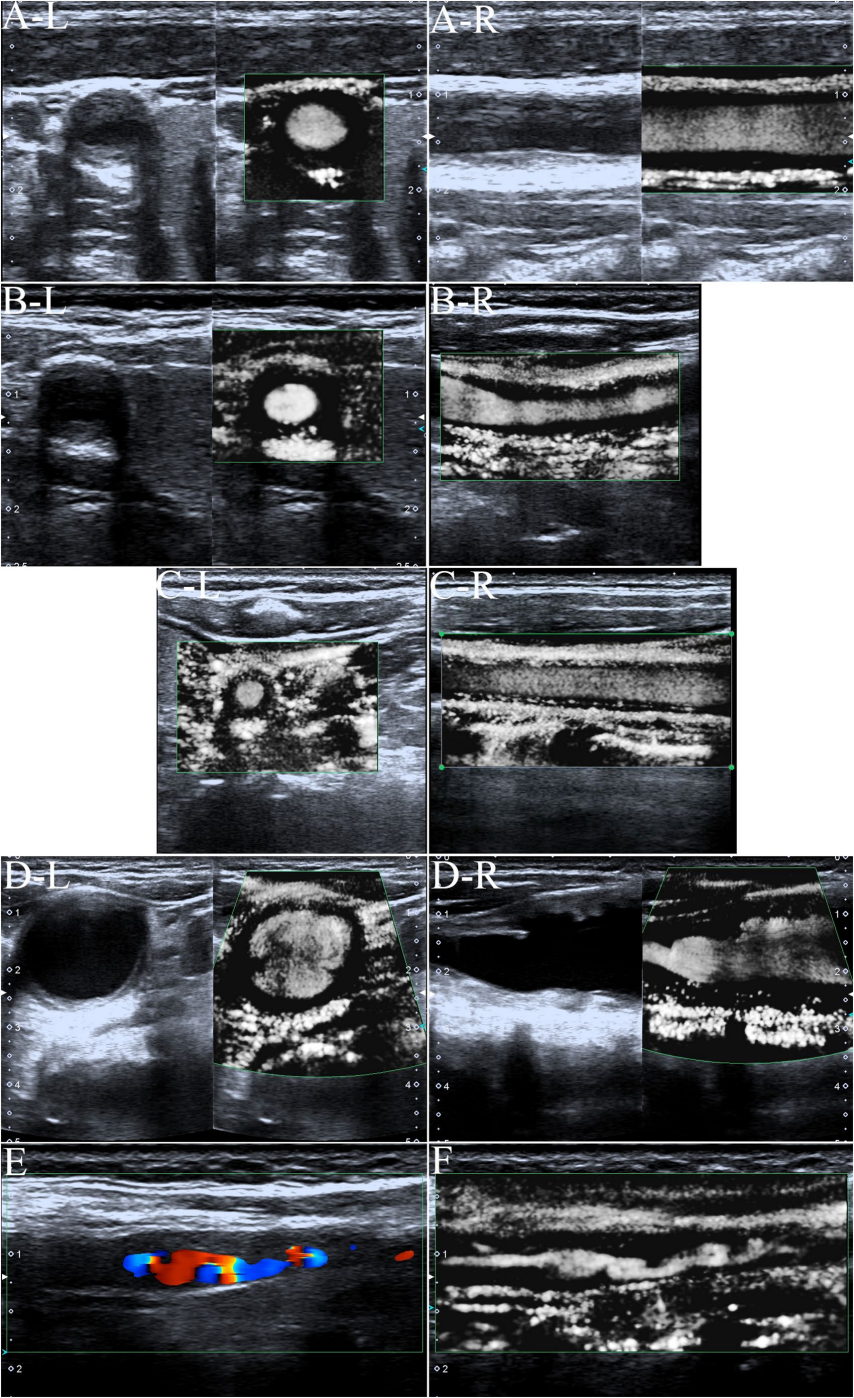
The superb microvascular imaging (SMI) of the carotid artery. **(A)** SMI G0, no visible short lines, dots, or short sticks of medium-high echogenic within the wall of the carotid artery (Left: Transverse section, Right: Longitudinal section). The asterisk marks the bloodstream in the carotid artery canal. The arrow shows the thickened wall of the carotid artery. **(B)** SMI G1, moderate visible short lines, dots, or short sticks of medium-high echogenic within the wall. The arrow shows neovessels in the thickened wall of the carotid artery (Left: Transverse section, right: Longitudinal section). **(C)** SMI G2, extensive visible short lines, dots, or short sticks of medium-high echogenic within the wall of the carotid artery. The arrow shows neovessels in the thickened wall of the carotid artery (Left: Transverse section, right: Longitudinal section). **(D)** Carotid lumen expansion and aneurysm formation (Left: Transverse section, Right: Longitudinal section). The asterisk marks the bloodstream in the carotid artery canal. The arrow shows neovessels in the thickened wall of the carotid artery aneurysm. **(E)** Color Doppler flow imaging showing carotid stenosis and segmental occlusion. **(F)** In contrast with **(E)**, SMI was able to clearly show low blood flow signals in the same carotid stenosis and segmental occlusion cavities.

**Figure 2:**
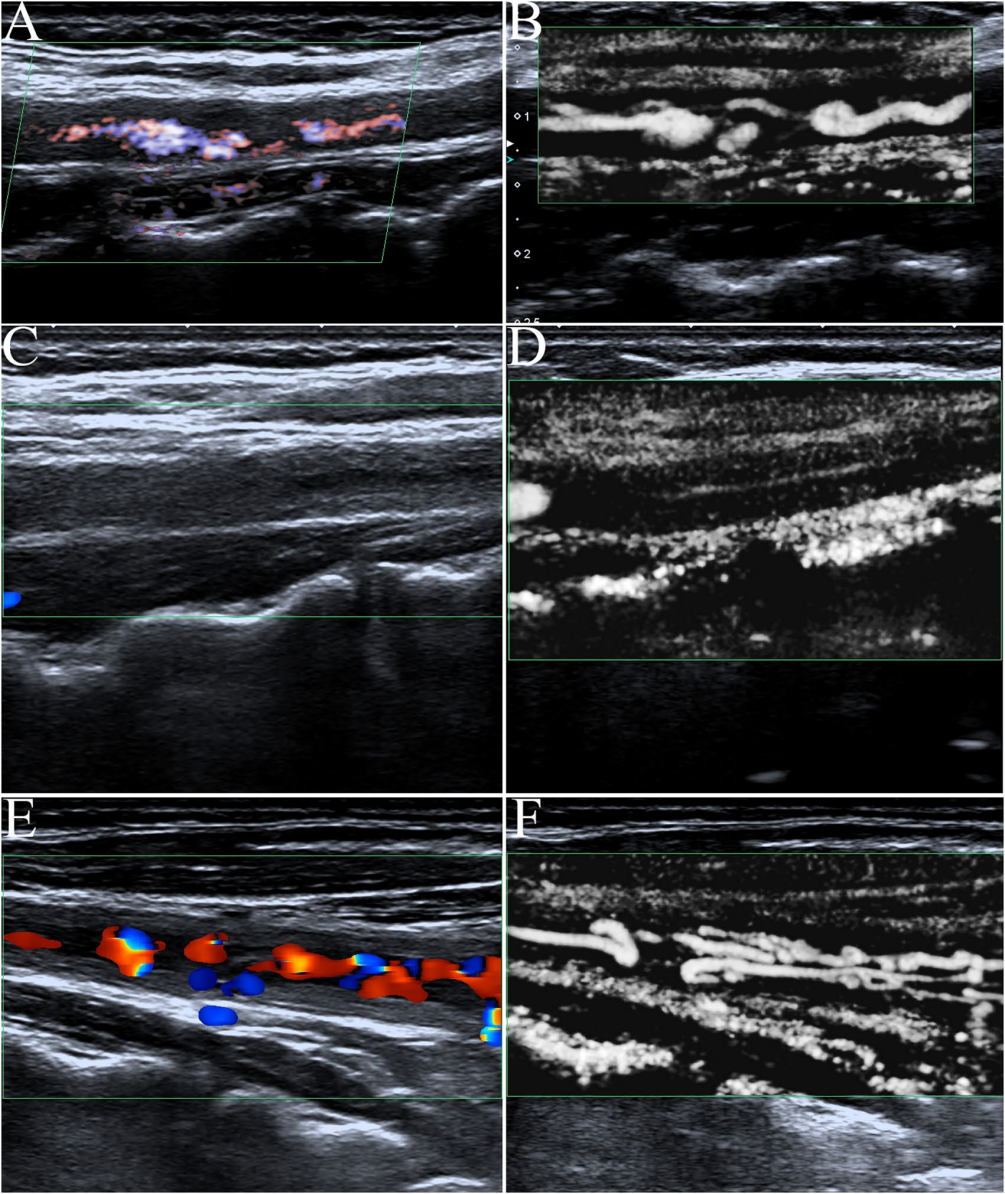
The contrast of color Doppler ultrasonography and SMI in blood flow display. **(A)** Advanced dramatic flow imaging showing carotid stenosis. **(B)** In contrast with **(A)**, SMI was able to clearly show low blood flow signals in the same carotid stenosis cavities. SMI improved image quality with a greater enhancement of vessel wall lumen and higher definition of the borders of the vascular lesion. **(C)** The affected carotid artery wall had varying degrees of diffuse thickening like macaroni signs, and color Doppler flow imaging showed occlusion of the carotid lumen. **(D)** In contrast with **(C)**, SMI could display very low blood flow signals in the lumen of the occluded carotid (fine lines) that color Doppler could not. **(E)** Color Doppler flow imaging showed intermittent, meandering, tortuous, and narrow blood flow signals in a fuzzy carotid lumen. **(F)** In contrast with **(C)**, SMI could clearly show the again unobstructed, meandering, tortuous, and narrow blood flow signals in the thickened vessel walls of the same carotid artery as twining wool.

Artery walls with concentric thickening, narrowing of the vascular diameter, and stenosis or occlusion of the lumen could be observed by carotid ultrasound (Figures 1, 2). In addition, carotid arterial aneurysms were readily observed (Figure 1D). SMI was able to display low blood flow signals in the lumen (Figures 1E,F, 2C,D) and concentric thickened walls of affected carotids (Figures 1B–D) that color Doppler could not. SMI can observe the collateral flow (the meandering, twisted, narrow blood flow signals) in the thickened vessel walls (Figures 2E,F). SMI improved image quality, enhanced the vessel wall and lumen, and gave a better definition of the borders of the vascular lesion (Figures 2A,B). Furthermore, we observed moving bright spots and linear flow within the vascular lesions that were principally visualized on the adventitial side of the vessels (Figure 1C), and we believe these signals represent neovessels of the neovascularization that could be visualized by SMI, only 55% could be visualized using color Doppler.

## Discussion

In this study, we investigated 96 patients to study the carotid SMI in evaluating the activity of TAK. The statistical analysis showed that the SMI and ESR had significant difference between the active and inactive groups. The median of SMI and ESR in the active group were significantly higher than those in the inactive group. And more importantly, SMI was an independent risk factor for TAK activity(OR = 4.778), indicating it as a potential marker of disease activity for patients.

TAK is a chronic, nonspecific, large vessel vasculitis with a global distribution but increased incidence in Asian ethnicities and higher morbidity in Asia (2, 14). It has been proposed that cellular immunity directed against vascular endothelial cells drives the inflammatory damage to large vessels, resulting in wall thickening, stenosis, occlusions, dilatations, and aneurysms (15). The newly formed feeding arteries in the wall of the diseased segment are closely related to the inflammatory response in the active stage (3). Whether TAK is in the active stage or not is important for clinical treatment. The active stage means that clinical treatment should be provided to control the inflammatory process. Vessel wall changes in inflammatory diseases could be evaluated using arterial angiography, computerized tomography (CT) scan, MRI, positron emission tomography (PET), and combined PET–CT scan (16). Higher disease activity in TAK does not translate into more severe vascular damage (17). However, previous imaging examinations focused more on evaluating the extent of vascular damage and involvement, and ignored the neovascularization in active inflammatory lesions tube wall. Using Duplex ultrasonography means that the resolution is theoretically superior, at least at high ultrasound frequencies (18–20). Duplex ultrasonography may be used for frequent controls due to ease of use, lack of radiation, and low costs.

SMI is a new ultrasound imaging technology developed based on color Doppler. It uses intelligent filtering technology, only filters out clutter, identifies and retains small blood vessels (diameter ≥ 0.1 mm) at very low velocities (minimum 0.8 cm/s), and is almost unaffected by angle. In the case of low velocity and high gain, it can still maintain the characteristics of less blood overflow and show the microvascular structure more clearly and completely. The existing Doppler modes are unable to distinguish motion artifacts from actual blood flow. With SMI, it is possible to analyze the characteristics of such motion artifacts and extract only the clinically relevant information (13). Compared with conventional Doppler techniques, SMI offers better detail resolution, faster frame rates, less clutter, and fewer flash artifacts (5, 12). In this study, the ability of color Doppler to display microvessels in the lesion was significantly lower than that of SMI.

Contrast-enhanced ultrasound can dynamically display the microvascular perfusion information of tissues in real-time, which is the “gold standard” for displaying the microvessels flow of organs. However, CEUS can only show the blood flow of the lesion during the filling time of the contrast agent (about 5–8 min), and there is a very short time limit for the filling and regression of the contrast agent. Therefore, CEUS cannot comprehensively display the microflow of extensive diffuse lesions in a sufficient time. SMI examination without the need for a contrast medium is not limited by filling time and phase, which ensures a detailed and comprehensive observation of the microflow of all diseased vessel walls. Moreover, SMI is significantly more economical and feasible than CEUS.

SMI is increasingly being used for vascular imaging indications (21, 22). In this study, our ability to detect neovascularization in the thickened vessel wall of patients with TAK using SMI technology was similar to previous reports (Magnoni M, Schinkel AF) that used contrast-enhanced ultrasound (3, 4). Currently, ESR and hsCPR are often used to assess aortic disease activity, but studies have shown that ESR and hsCPR can be normal in patients in the active stage. Therefore, a normal ESR does not represent the extinction of vascular wall inflammation and the cessation of lesions, and both values are non-specific markers of inflammation, lacking specificity for the evaluation of vascular inflammation (23–26). Studies have shown that the evaluation of vascular inflammation using CEUS is more sensitive than acute phase reactants (27). The results of our study showed that feeding vessels in the wall of the involved carotid artery detected using SMI were an independent risk factor for predicting the active stage of TAK. This is consistent with the pathophysiological changes of proliferation and expansion of nutrient vessels in active TAK and the results of previous studies (28–30).

In Sato W’s study, arterial wall vascularization detected using SMI predicted the fluorodeoxyglucose (FDG) uptake with 100% sensitivity, 97% specificity, 83% positive predictive value, and 100% negative predictive value (*n* = 9,54 sites). Therefore, it was concluded that carotid artery hypervascularity detected using SMI is a potential marker of disease activity in patients with TAK (31). In our study, the SMI G1 and G2 were used as cut-off values to diagnose active TAK. The diagnostic efficacy was lower than that of the above studies because the control criterion was clinical NIH ≥ 2 scores, rather than the FDG uptake. In theory, SMI is consistent with the FDG uptake in detecting vessel wall vascularization. SMI allows the dynamic assessment of carotid wall vascularization, which is a potential marker of disease activity in patients with TAK.

This study has several limitations. First，the SMI technique used to evaluate neovascularization in the vessel walls of patients with TAK was not compared to blank control and other gold standard methods. MRI or contrast-enhanced ultrasound should be used for direct comparison with SMI in future studies. In addition, SMI-based diagnosis of vessel wall neovascularization was semi-quantitative instead of quantitative, which introduced subjectivity to the data analysis pipeline. The sensitivity and specificity concluded from this small sample-size study remained further testify in clinic. Finally, the only SMI technology utilized was the Toshiba ultrasound system. However, SMI technology can indeed detect the proliferation of tiny vessels with smaller inner diameter and slower flow that color Doppler cannot detect.

## Conclusion

In summary, SMI was an independent risk factor for TAK activity, indicating it as a potential marker of disease activity for patients with TAK.

## Data availability statement

The original contributions presented in the study are included in the article/supplementary material, further inquiries can be directed to the corresponding author.

## Ethics statement

The studies involving human participants were reviewed and approved by the Ethics Committee of Beijing Anzhen Hospital. The patients/participants provided their written informed consent to participate in this study.

## Author contributions

F-JL and W-PC: conception and design of the research, analysis and interpretation of the data, and critical revision of the manuscript for intellectual content. F-JL and YC: acquisition of data and statistical analysis. F-JL: writing of the manuscript. All authors contributed to the article and approved the submitted version.

## Conflict of interest

The authors declare that the research was conducted in the absence of any commercial or financial relationships that could be construed as a potential conflict of interest.

## Publisher’s note

All claims expressed in this article are solely those of the authors and do not necessarily represent those of their affiliated organizations, or those of the publisher, the editors and the reviewers. Any product that may be evaluated in this article, or claim that may be made by its manufacturer, is not guaranteed or endorsed by the publisher.

## Abbreviations

SMI: Superb microvascular imaging
TAK: Takayasu’s arteritis
ANOVA: One-way analysis of variance
ESR: Erythrocyte sedimentation rate
CEUS: Contrast-enhanced ultrasound
MRI: Magnetic resonance imaging
hsCRP: High-sensitivity C-reactive protein
NIH: National Institutes of Health
SD: Standard deviation
